# Evasion of Immunological Memory by *S. aureus* Infection: Implications for Vaccine Design

**DOI:** 10.3389/fimmu.2021.633672

**Published:** 2021-02-22

**Authors:** Omid Teymournejad, Christopher P. Montgomery

**Affiliations:** ^1^Center for Microbial Pathogenesis, Abigail Wexner Research Institute at Nationwide Children's Hospital, Columbus, OH, United States; ^2^Department of Pediatrics, The Ohio State University College of Medicine, Columbus, OH, United States

**Keywords:** *S. aureus*, vaccine, T cell, antigen presenting cell (APC), human leukocyte antigen (HLA)

## Abstract

Recurrent *S. aureus* infections are common, suggesting that natural immune responses are not protective. All candidate vaccines tested thus far have failed to protect against *S. aureus* infections, highlighting an urgent need to better understand the mechanisms by which the bacterium interacts with the host immune system to evade or prevent protective immunity. Although there is evidence in murine models that both cellular and humoral immune responses are important for protection against *S. aureus*, human studies suggest that T cells are critical in determining susceptibility to infection. This review will use an “anatomic” approach to systematically outline the steps necessary in generating a T cell-mediated immune response against *S. aureus*. Through the processes of bacterial uptake by antigen presenting cells, processing and presentation of antigens to T cells, and differentiation and proliferation of memory and effector T cell subsets, the ability of *S. aureus* to evade or inhibit each step of the T-cell mediated response will be reviewed. We hypothesize that these interactions result in the redirection of immune responses away from protective antigens, thereby precluding the establishment of “natural” memory and potentially inhibiting the efficacy of vaccination. It is anticipated that this approach will reveal important implications for future design of vaccines to prevent these infections.

## Introduction

*Staphylococcus aureus* is an aerobic gram-positive organism that can cause local and systemic infections in humans, ranging in severity from skin and soft tissue infection (SSTI) to more invasive infections such as osteomyelitis, septic arthritis, pneumonia, bacteremia, and septic shock (1). 20–80% of humans are colonized with *S. aureus* in the nasopharynx, skin, and/or gastrointestinal tract, providing a reservoir for subsequent infection and transmission (2, 3). A major issue in the field is that “natural” immune responses against *S. aureus* infection do not seem to be protective and recurrent infection is common—roughly 50% of adults and children with SSTI have a recurrence within a year (4, 5). Developing an effective vaccine has been challenging; all candidate vaccines tested thus far have failed to protect against *S. aureus* (6–8). These failures must be considered in the context of nearly ubiquitous exposure to *S. aureus*; it is accepted that most individuals are exposed to *S. aureus* shortly after birth and throughout childhood (9). This is reflected in the fact that most people, regardless of age or history of symptomatic infection, have detectable levels of anti-staphylococcal antibodies (9). However, whether these antibodies are protective remains elusive. Although there is evidence in murine models that both cellular and humoral immune responses are important for protection against *S. aureus*, human studies suggest that T cells are most important in determining susceptibility to infection (10, 11).

## An “Anatomic” Approach to Understanding *S. aureus* Evasion of Adaptive Immunity

**Figure 1:**
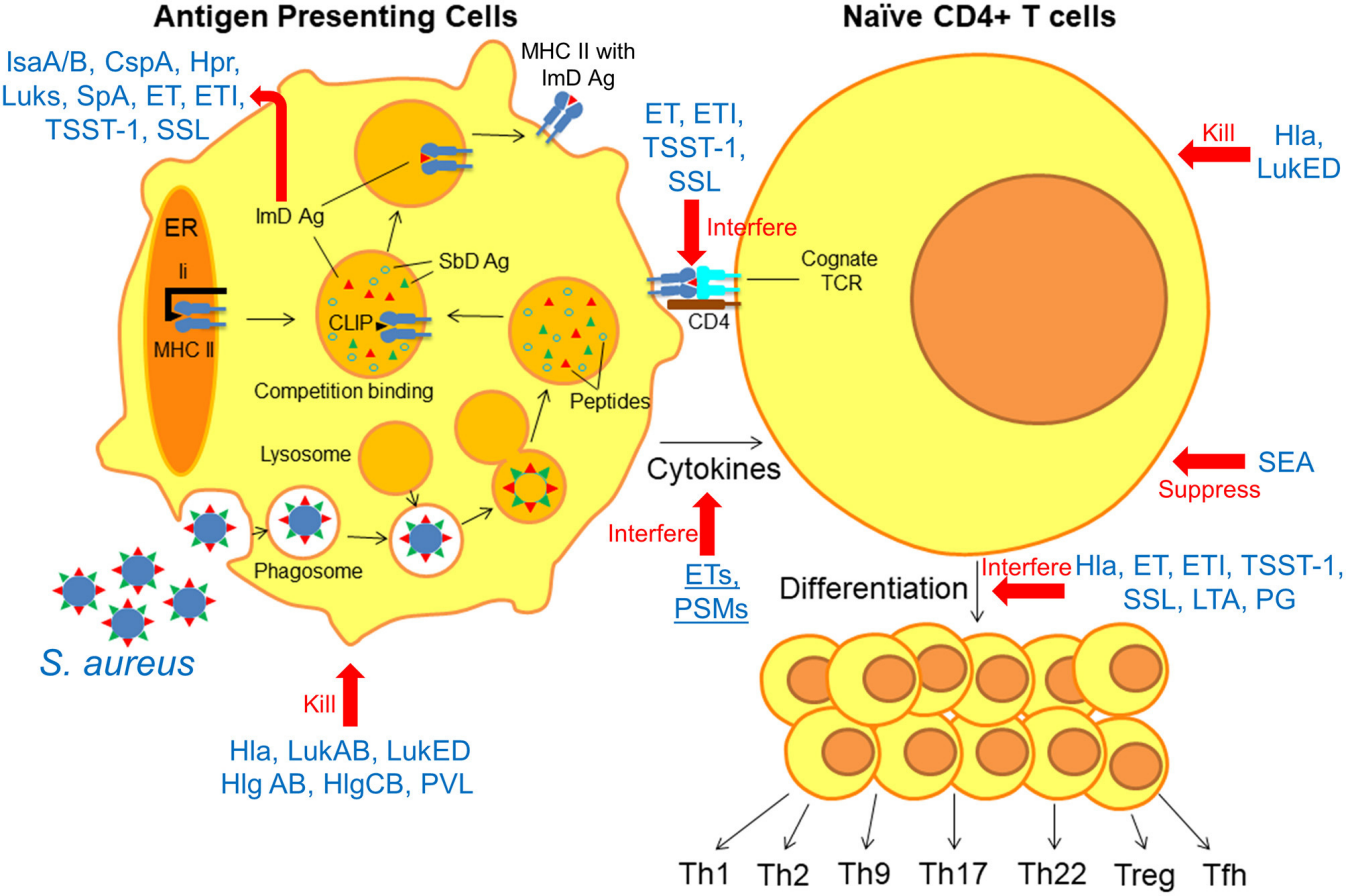
Anatomy of the interfering *S. aureus* virulence factors with antigen presentation and T cell differentiation. Following phagocytosis of *S. aureus* by APCs, bacterial antigens are “processed” into epitopes by proteolysis. Immunodominant (ImD) epitopes bind to MHC II based on binding affinity and are trafficked to the cell surface, where they “find” cognate TCRs on naïve T cells. Based on these model, there is a competition between different staphylococcal antigens for binding to MHC II proteins, and ImD antigens such as IsaA/B, CspA, Hpr, Luks, SpA, ET, ETI, TSST-1, and SSL are more successful in this competition. However, non-protective ImD antigens can “outcompete” protective subdominant (SbD) antigens. *S. aureus* can interfere with the antigen presentation and T cell differentiation in multiple steps; Hla, LukAB, LukED, Hlg AB, HlgCB, and PVL directly kill APCs and T cells. ET, ETI, TSST-1, and SSL as superantigens interfere with peptide-MHC II and TCR interaction. ETs and PSMs interfere with APC cytokines production. SEA, Hla, ET, ETI, TSST-1, SSL, LTA, and PG suppress T cell activation and interfere with T cell differentiation.

Herein, we take a systematic approach toward identifying knowledge gaps in our understanding of protective adaptive immunity against *S. aureus* by reviewing the “anatomy” of the immune response. We focus on current knowledge of how anti-staphylococcal immune responses are generated at each step of the process, and how *S. aureus* can evade or interfere with these processes. During infection, antigen presenting cells (APCs) phagocytose bacteria and “process” them into smaller peptides by proteolysis (Figure 1) (12). These peptides, called epitopes, may then bind to Major Histocompatibility Complex (MHC) proteins depending on the specific binding affinity of each peptide for the MHC proteins (13). Epitope-bound MHC proteins are then trafficked to the surface of the APCs, where they are presented to cognate T cell receptors (TCR) on naïve T cells within secondary lymphoid organs (MHC Class I for CD8^+^ T cells, MHC Class II for CD4^+^ T cells) (14). Binding of the epitope-MHC complex to its cognate T cell receptor on naïve T cells results in differentiation into one of a number of T cell subsets, depending on the local inflammatory milieu and cytokines expressed by innate immune cells (15). These T cell subsets include both effector and memory T cell populations, the latter of which is responsible for the establishment of immunological memory (15). Based on accumulated evidence regarding the importance of T cell responses in defense against *S. aueus* infection, this review will focus primarily on CD4^+^ T cell responses in the context of protective adaptive immunity. It is anticipated that this approach will reveal important implications for future design of vaccines to prevent these important infections.

## *S. aureus* and Antigen Presenting Cells

### Overview

APCs activate T cells to shape immunological memory. Professional APCs include dendritic cells, macrophages, and B cells and are located in a variety of tissues. Dendritic cells are present in the skin (Langerhans cells) and the lining of the nose, lungs, stomach, and intestines (16). Macrophages, primarily differentiated from peripheral blood monocytes, are found in many tissues (17). B cells are produced in the bone marrow and migrate to the spleen and other secondary lymphoid tissues for maturation (18). APCs promote adaptive immune response by secreting cytokines and by presenting specific epitopes bound to MHC proteins. APCs provide three signals to stimulate CD4^+^ T cells; peptide-MHC II complex, co-stimulatory molecules such as B7.1 and B7.2, and stimulatory cytokines such as IL-12 (19). During infection, *S. aureus* manipulates these signals to evade host immune responses (20).

### Manipulation of APC Cytokine Secretion

Generally, activation of human and mouse DCs results in secretion of IL-12, which in turn promotes Th1 immune responses. Th1 cells secrete IFNγ, a cytokine that activates macrophages at the site of infection to clear pathogens (21). Moreover, stimulation of epidermal DCs (Langerhans cells) results in secretion of the proinflammatory cytokine IL-6 and IL-12 and inhibition of TRAC, a cytokine that promotes Th2 responses (22). Several *S. aureus* virulence factors impact APC cytokine secretion (23). For example, *S. aureus* enterotoxin B induces production of high levels of TNF-α and low levels of IL-12 in DCs (24). In mice, depletion of DCs prior to *S. aureus* infection resulted in higher lethality accompanied by higher bacterial burdens in the kidneys and lungs (25). This was concluded to be secondary to inhibition of IL-12 production because protection was restored by injection of recombinant IL-12. Similarly, phenol-soluble-modulins (PSMs) produced by CA-MRSA strains upregulate CCR7 on the surface of DC subsets and stimulate IL-10 secretion, while inhibiting TNF production (26). Together, these findings suggest that *S. aureus* can have tolerigenic effects of DCs. In contrast, *S. aureus* induces production of high levels of IL-12 and IL-23 by monocytes, monocyte-derived macrophages, and DCs, resulting in robust Th1 (IFNγ) and Th17 (IL-17) responses (27). These opposing data suggest that *S. aureus* can elicit protective or inhibitory responses in APCs, depending on expression of specific virulence factors and the local milieu.

### Toxin-Mediated Killing of APCs

A major mechanism by which *S. aureus* may interfere with APC function is by toxin-mediated APC killing. *S. aureus* produces a number of bi-component pore-forming leukotoxins that directly kill APCs by creating channels in the plasma membrane (28). Bicomponent toxins are comprised of two subunits, called S (slow) and F (fast), that oligomerize on the surface of target cells to form membrane-spanning pores (29). *S. aureus* strains isolated from humans produce at least four leukotoxins; the Panton-Valentine Leukocidin (PVL), gamma (γ)-hemolysin (HlgACB), Leukotoxin ED (LukED), and Leukotoxin AB/GH (LukAB/GH) (30). Each leukotoxin has distinct cellular targets that are defined by receptor-specific interactions; monocytes, macrophages, and DCs are targeted by LukAB (CD11b), LukED (CCR5, CXCR1, CXCR2), Hlg AB (CCR2, CXCR1, CXCR2), HlgCB (C5aR1, C5aR2), and PVL (C5aR1, C5aR2)(29). In the context of this review, the direct toxicity of leukotoxins is particularly noteworthy because APCs are an essential link between innate and adaptive immunity (18).

α- toxin (Hla) is a small β-barrel toxin that oligerimizes to form pores in host cell membranes, resulting in cell lysis and death by osmotic swelling and rupture (31). Hla binds to its cellular receptor, ADAM10 (32), resulting in toxicity toward a wide range of mammalian immune cells, including T cells, monocytes, dendritic cells, macrophages, and neutrophils (33). Therefore, similar to the bicomponent leukotoxins, Hla expression can disrupt antigen presentation to T cells by directly killing APCs and by inhibiting differentiation of T cells to effector and memory cells (34). Primary infection in C57BL/6 mice with Hla-producing *S. aureus* impaired protection against recurrent infection (35). This was attributed, at least in part, to direct toxicity of Hla to dendritic cells, whose numbers were decreased following skin infection with wild type *S. aureus*, but not an Hla mutant (35). Consistent with this notion, anti-Hla IgG protects against necrosis in the skin and lungs (36, 37), but the effects of antibody on immune cell toxicity are not yet clear and may depend on the site of infection (37). Along these lines, passive transfer of anti-Hla antibody into mice protected against dermonecrosis by neutralizing toxin, rather than by enhancing opsonophagocytosis (38). In this model, Hla-specific antibody also protected against toxicity toward dermal monocytes/macrophages. Therefore, while it is tempting to speculate that Hla-specific IgG protects in part by inhibiting toxicity toward APCs, further elucidation of the mechanisms of protection is necessary. Taken together, these findings demonstrate that *S. aureus* toxins can directly kill APCs, but the importance of these processes in disturbing adaptive immune responses has yet to be conclusively demonstrated in the clinical setting.

## Presentation of *S. aureus*-Specific Epitopes by APCs

### Overview

Once APCs internalize organisms, proteins are cleaved to small peptides, which are bound to MHC and trafficked to the cell surface for presentation to cognate TCRs on naïve T cells (14). Presentation of specific epitopes is highly dependent on binding to MHC, which is dependent on the affinity of peptide-MHC binding. Peptides that are bound strongly to MHC are more “available” for presentation. These are called immunodominant (ImD) peptides, and this step is critical for determining the epitopes against which the immune response is focused. In contrast, peptides with a low affinity for MHC are less efficiently presented, and are termed subdominant (SbD) epitopes (39). ImD epitopes may also be determined by the affinity of peptide-MHC presented epitopes to bind to their cognate T cell receptors (TCRs). The selection of epitopes that drive ImD/SbD antibody responses is somewhat different. For example, un-processed antigens should be accessible to B cell receptors (BCR). After processing by B cells and presentation of epitopes to helper T cells, B cells will be activated against specific epitopes, subsequently followed by antibody affinity maturation and isotype switching. Therefore, antibodies develop against the antigens that are both “available” to BCRs and bind BCRs with strong affinity (40). One of the challenges in establishing immunological memory against *S. aureus* is that the staphylococcal ImD peptides may not elicit protective memory T cell and antibody responses. In this scenario, one can envision that a strong response against non-protective ImD epitopes may be generated at the expense of a response against more protective, but SbD, epitopes. Therefore, identification of ImD epitopes is critical to better understand how natural immune responses develop.

### Immunodominant *S. aureus* Antigens that Drive Antibody Responses

In addition to establishing immunological memory against *S. aureus*, T cells work in concert with B cells resulting in high affinity antibody production. A durable and effective antibody response requires T helper cells to assist B cells for antibody affinity maturation and isotype switching. Clearly, quantification of antibody levels is simpler and more reproducible than T cell responses. Therefore, the majority of studies on *S. aureus*-specific immunity to date have focused on antibody levels. While a detailed review of antibody levels in children and adults with *S. aureus* infection is beyond the scope of this review, a few key observations have emerged. First, children develop antibody responses during the first year of life and the antibody levels increase throughout childhood (41, 42). Second, high levels of antibodies against selected *S. aureus* antigens are stable for years in healthy individuals and appear to be functional (41). However, despite high antibody levels during childhood, there may be diminished ability of antibodies to neutralize critical *S. aureus* toxins (42). Third, children with *S. aureus* infections generally have higher antibody levels, compared with healthy children (41, 43).

Because antibody levels are more readily quantifiable, compared with T cell responses, one approach is to extrapolate immunodominant antigens from the many antibody studies reported. For example, Lorenz et al. identified four immunodominant proteins during *S. aureus* infection; IsaA, IsaB, CspA and Hpr (44). Although healthy *S. aureus* carriers had significantly higher levels of IgG against IsaA comparing to non-carriers, active immunization against IsaA is not protective in mice and anti-IsaA levels are not correlated with protection against *S. aureus* infection (45). Antibody levels against LukS, LukE, HlgA, HlgC, LukF, LukD, HlgB, Hla, and Hla were high in children with *S. aureus* infection, compared with healthy controls (46). Importantly, antibody levels correlate with antigen-specific circulating memory B cells (47). Radke et al. identified ImD antigens using a proteomic approach to quantify antibody levels against over 2600 *S. aureus* antigens. They identified 104 proteins against which all patients had high-level reactivity. All of the above-mentioned ImD proteins are reported within top fifty highly reactive proteins, suggesting some level of conservation of ImD antigens within the population (48).

### Immunodominant T Cell Antigens in *S. aureus*

Unfortunately, epitopes that are ImD in driving B cell/antibody responses are not necessarily the same epitope that drive ImD T cell responses. A variety of ImD T cell epitopes have been identified in animal models, including epitopes within a phosphodiesterase (Plc) (49), LukE and LukS-PV (50), nuclease (51), and IsdB (52), and clumping factor A and protein A appear to elicit T cell responses in both mice and humans (53, 54). However, unlike antibody responses, a relative hierarachy for T cell antigens/epitopes has not been established. To address this, Kolata et al. treated PBMCs from healthy adults with conserved extracellular proteins of *S. aureus* that elicit an antibody response in most individuals, including the lipase Geh, the phosphodiesterase GlpQ, the phospholipase Plc, and Hla. They observed that the strongest responses were specific for Hla and they found high frequencies of Hla-specific proliferating T cells, compared with the other proteins tested (55). Since Hla also elicits ImD antibody responses, this may suggest that the same antigens drive ImD antibody and T cell responses. However, it is likely that different epitopes within each antigen separately drive antibody and T cell responses. In the future, functional studies should be complemented by *in silico* approaches that use structure-based algorithms to predict ImD T cell epitopes (49, 56).

### Protective vs. ImD Responses

Since individuals are exposed to *S. aureus* quickly after birth, it is conceivable that immunological memory develops primarily against non-protective ImD antigens. In this scenario, pre-existing memory against non-protective ImD antigens may inhibit vaccination later in life. This is reminiscent of Francis's theory of Original Antigenic Sin (OAS) (57, 58). In OAS, sequential exposure to antigen variants induces a preferential antibody response to an antigen encountered in the past. Consequently, the immune response to the current antigen is weaker (59). However, there remains no compelling evidence that OAS mechanisms are operant in *S. aureus*-specific immunity. Another model of immune imprinting that describes how ongoing exposure to pathogens may reinforce immune responses against ImD antigens is called “antigenic seniority.” In contrast to OAS, in which patterned immune responses are assumed to be disadvantageous, antigenic seniority describes a process in which early life exposures “build the framework for a hierarchy of immune responses” (40). In this context, ImD responses that are elicited early in life are thought to have a “senior” or privileged position, but subsequent exposures, while boosting these responses, may also produce responses against other SbD antigens. There is some evidence for these mechanisms in the development of *S. aureus*-specific immunity. For example, Pelzek et al. demonstrated that adults with *S. aureus* SSTI infection have a diverse set of antigen-specific memory B cells, and these memory B cells correlate well with antigen-specific antibody levels (47). However, much of the antibody response was directed toward cross-reactive antibodies that recognized multiple leukotoxins. Importantly, despite the presence of memory B cells, they did not observe significant increases in antigen-reactive antibody-secreting plasmablasts and plasma cells during infection. Similarly, they found increased memory B cell frequencies only for certain antigens.

Together, these findings suggest a clonal response focused on a limited number of cross-reactive epitopes and provide evidence for a patterned B cell response that limits the diversity of the immune response. A potentially similar mechanism was elucidated by Pauli et al. They found that staphylococcal protein A (SpA) polarized plasmablast responses away from other antigens, suggesting that SpA acts as an ImD antigen in limiting responses against other, potentially protective, antigens (60). Pre-existing natural antibodies may also “mask” protective but SbD epitopes (40), thereby precluding the ability of exposure to these antigens to elicit protective responses. This may be of particular relevance given the broad range of antigen-specific antibodies observed in individuals with *S. aureus* infection (48). While we do not yet know whether similar mechanisms might inhibit the diversity of T cell responses during *S. aureus* infection, it is of interest that similar mechanisms have been a challenge for influenza vaccination, in which past exposure may shape the immune system such that vaccination may reinforce responses to epitopes from past exposures, rather than those targeted by the current vaccine (61). We hypothesize that natural immune responses directed against non-protective staphylococcal ImD antigens result in a phenomenon similar to these models. If this is the case, natural exposure to *S. aureus* may pattern a non-protective memory response over a lifetime, which is not able to prevent re-infection and may even interfere with subsequent vaccine attempts later in life. However, much work needs to be done to test this hypothesis. For example, identification of antibody and T cell responses that predict protection against *S. aureus* infection must be prioritized in order to move forward (4). In the context of vaccines, mechanistic studies that use “pre-exposed” rather than naïve mice would prove informative and may better simulate vaccination of a human population.

### Role of MHC Haplotypes

There is considerable heterogeneity of HLA (Human Leukocyte Antigen) /MHC haplotypes in the human population, and certain haplotypes have been associated with susceptibility to a number of infections. For example, associations between specific HLA Class II polymorphisms and susceptibility to HIV infection, hepatitis, leprosy, tuberculosis, malaria, leishmaniasis, and schistosomiasis have been reported (62). Consistent with this notion, there is an association between HLA Class II gene polymorphisms and susceptibility to *S. aureus* infection in white and African-American populations (63, 64). Mouse models have uncovered one possible mechanistic explanation for these observations. BALB/c mice are protected against secondary SSTI, but C57BL/6 mice are not (50). These divergent phenotypes were explained by the different MHC class II haplotypes in the mouse strains: BALB/c mice express MHC H-2^d^ and C57BL/6 express H-2^b^ (50). In this model, antibody responses against Hla and Th17 responses against LukE and LukS-PV were observed only in mice that express MHC H-2^d^. Moreover, concomitant infection inhibited vaccine efficacy in C57BL/6 mice, but not BALB/c mice. The mechanism of this inhibition was due to strong binding of protective epitopes to MHC H-2^d^, but not H-2^b^ (50). Based on these findings, a model emerges of competition between different staphylococcal antigens for binding to MHC proteins, and ImD antigens are more successful in this competition. However, non-protective ImD antigens can “outcompete” protective antigens, depending on the host genetic background (50). Fortunately, vaccination of naïve mice expressing either H-2^d^ or H-2^b^ was effective. However, these findings have not been translated to human infection.

## Nonspecific T Cell Activation: *S. aureus* Superantigens

Another mechanism by which *S. aureus* can inhibit protective T cell responses is by expression of superantigens. More than 20 superantigens have been identified in different strains of *S. aureus*. Approximately 80% of *S. aureus* isolates from infected patients harbor at least one superantigen, although most isolates express more than one (65). Staphylococcal superantigens are classified as Enterotoxins (ETs), Enterotoxin like proteins (ETls), Toxic Shock Syndrome Toxin−1 (TSST-1) and staphylococcal superantigen-like proteins (SSL) (66). Conventional T cell responses are mediated by the interaction of antigens with hypervariable regions of the αβ T cell receptor (TCR). As such, conventional antigens can stimulate ~0.01% of naïve T cells due to the diversity of CDR3 (Complementarity-determining region 3) in T cell receptors. In contrast, superantigens do not bind to CDR3, but instead bind T cells via a TCR β-chain variable domain (Vβ)-dependent mechanism (65) Because there are limited numbers of functional Vβ regions (around 50) in humans, superantigens can activate many T cells with different TCR (65, 67). Furthermore, superantigens can activate T cells much more strongly than conventional antigens and they are able to activate up to 30% of the T cell pool in picogram concentrations (67).

The consequences of superantigen expression on development of adaptive immunity remain to be fully elucidated. Staphylococcal superantigens are unique in that they activate T cell responses to evade host immunity (68). One mechanism by which superantigens impair cellular memory is by interfering with signaling through the TCR and induction of clonal tolerance (anergy) (69). For example, following stimulation of PBMCs by staphylococcal Enterotoxin C1, CD25^+^ FoxP3^+^ regulatory T cells proliferated and secreted the immunosuppressive cytokine IL-10 (70). Because responses against staphylococcal superantigens are highly immunodominant, there has been considerable interest in pursuing a superantigen vaccine (71–73). Unfortunately, none has yet proven effective in clinical studies.

## Expansion of NAÏVE to Memory and Effector T Cells in *S. aureus* Infection

### Overview

T cells circulate in blood, lymph vessels, and secondary and peripheral lymphoid tissues. Once mature naive T cells migrate from the thymus to secondary lymphoid organs (lymph nodes, spleen or MALT (Mucosa-associated lymphoid tissues), the interaction between peptide-loaded MHC on the surface of APCs and a cognate TCR results in activation and subsequent differentiation to effecter or memory cells (74, 75). There are various subsets of T cells with different functions in host immunity whose differentiation from naïve T cells depends upon distinct cues (e.g., different cytokines, MHC Class II-peptide complex, and costimulatory signals). Naïve CD4^+^ T cells can differentiate into several subsets, including Th1, Th2, Th9, Th17, Th22, Treg and Tfh. The classical distinction among these subsets is simplified and many of these cells may have characteristics of one or more subsets and they also may retain plasticity (76). For example, following presentation of their cognate epitope, naïve CD4^+^ T cells differentiate to Th1 cells following stimulation with IL-12 and IFNγ, to Th2 cells following stimulation by IL-4 and IL-2, or to Th17 cells in the presence of TGF-β and IL-6 (77). Depending on the tissue and the specific stimulus, activated T cells may return to the bloodstream and migrate to the sites of infection or inflammation in peripheral tissues (78).

### Importance of T Lymphocytes in Defense Against *S. aureus*

There is accumulated evidence that T cells response are critical for defense against *S. aureus* infection in humans and in experimental models. The importance of T cell subsets in defense against *S. aureus* has been the subject of several outstanding reviews, and will only be briefly discussed here (79, 80). For example, it is well-established that Th17 cells are important in defense against extracellular bacteria (such as *S. aureus*) via the production of a number of cytokines, resulting in neutrophil activation and recruitment to the site of infection (81). Individuals with hyper immunoglobulin E syndrome, classically caused by mutations in the DNA-binding domain of STAT3, have defects in pathways that result in Th17 cell differentiation and are highly susceptible to recurrent mucocutaneous *S. aureus* infections (82, 83). Individuals with poorly controlled HIV with low CD4^+^ counts are also susceptible to *S. aureus* infection (84). These studies are complemented by a number of animals studies demonstrating the importance of Th17/IL-17A mediated immunity (85–87). The role of Th1-mediated immunity is less clear, as several studies have demonstrated a protective role for this subset in mouse models, but several groups have also reported that Th1-mediated responses may also inhibit protective immunity (53, 88, 89). A role for Th2 responses has been established in allergic diseases mediated by *S. aureus* (90). While Th22 responses may complement Th17-mediated protection at the mucocutaneous interface (91), the role of this subset is less well-defined. γδ T cells, which display neither CD4 nor CD8 markers on their surface, are a major source of IL-17 production in mouse models (92), but may be more polarized toward IFNγ secretion in humans (93).

### *S. aureus* Toxins Kill T Cells

As alluded to earlier, a number of staphylococcal toxins are able to directly kill T cells. For example, Alonzo et al. showed that LukE binds to CCR5 on the surface of CD4^+^ T cells, resulting in oligomerization of LukE and LukD (94). This subsequently results in killing of CCR5^+^ T cells. In support of the importance of this process, CCR5-deficient mice are strongly protected from lethal *S. aureus* infection. Incubation of peripheral lymphocytes with LukED resulted in CCR5^+^ T cell depletion, most of which were effector memory T cells. Of note, CCR5 is expressed on both Th1 and Th17 subsets, suggesting a potential evasion strategy by which *S. aureus* directly kills IL-17 and IFN-γ-producing T cells. Similarly, Hla induces programmed cell death of human T cells during USA300 infection (95). In a mouse model, expression of Hla during primary infection results in abrogated memory T cell responses, at least in part due to direct toxicity on T cells (35). In comparison with wild-type *S. aureus*, infection with a Hla deletion mutant resulted in greater expansion of antigen- specific memory T cells. Interestingly, maternal immunization with Hla resulted in enhanced development of memory T cells in pups following post-natal infection, supporting the idea that early exposure to Hla interferes with the development of immunological memory (35). Bonifacius et al. have recently reported that Hla induced direct death of Th1-polarized cells, while Th17 cells were relatively resistant. They demonstrated that toxicity is independent of the Hla-ADAM10 interaction and is not due to differential activation of caspases. Instead, they suggested an increased susceptibility of Th1 cells toward Ca2^+^-mediated activation-induced cell death (96).

### Other Mechanisms by Which *S. aureus* Suppresses T Cells

Leech et al. demonstrated that Hla limits the expansion of tolerigenic Tregs (97). They showed that the number of Tregs in neonatal mice colonized with *S. aureus* is relatively low upon cutaneous re-exposure as adults and that colonization with an Hla mutant resulted in recovery of pathogen-specific Tregs. Interestingly, topical application of recombinant Hla during *S. epidermidis* colonization resulted in a lower percentage of *S. epidermidis-*specific Tregs, but whether this is due to direct toxicity toward Tregs remains to be determined. Other staphylococcal virulence factors also suppress T cell responses. For example, staphylococcal cell wall components such as lipoteichoic acid or O-acetylation of peptidoglycan suppressed T cell proliferation and polarization of Th cells to Th1 and Th17 (98, 99). Staphylococcal enterotoxin A (SEA) upregulated anergy-related genes in CD4^+^ T cells isolated from Atopic Dermitidis patients (100). *S. aureus* may also suppress T cell responses by eliciting the expansion of other suppressive immune cells. For example, *S. aureus* infection in mice resulted in expansion of granulocytic and monocytic Myeloid-Derived Suppressor Cells (MDSCs) (101). This expansion was accompanied by suppression of T cell responses. Taken together, these findings demonstrate that *S. aureus* is able to suppress T cells via multiple mechanisms.

## Challenges in Creating a Protective Vaccine

### Overview

The enormous burden of *S. aureus* infections and emerging antimicrobial resistance makes a vaccine to prevent these infections a worthy goal (102). Despite a lack of understanding of naturally-acquired immunity against *S. aureus*, several large vaccine tials have targeted adults populations with a high incidence of *S. aureus* infection (6). Unfortunately, despite promising protection in pre-clinical models, none that advanced to clinical trial has proven effective against human infection (103). Examples include the capsular proteins CP5/CP8 (StaphVAX, Nabi) in patients undergoing hemodialysis, the iron scavenger protein IsdB (V710, Merck) in patients underoing cardiac surgery, and a combination of capsular proteins, clumping factor A (ClfA), and a manganese transporter (MntC) in patients undergoing orthopedic surgery (SA4Ag, Pfizer) (104–106). In each case, vaccination failed to prevent infection despite high levels of elicited antibody in vaccine recipients. There are several possibilities to explain these failures, including high levels of pre-existing immunity among vaccine recipients, the antigens and preclinical models selected for evaluation, the exclusion of vaccine adjuvants, a lack of identified correlates of immunity, and the chosen target populations for vaccination.

### Antigen Selection and Preclinical Models

We believe that antigenic seniority may be an obstacle toward developing a successful vaccine against *S. aureus* infection. Because early life exposure by *S. aureus* so strongly influences the developing immune system, it is probable that this exposure not only prevents protective immunity, but may also inhibit subsequent vaccine efforts in older individuals (20, 61). If, as in influenza, antigenic seniority is a phenomenon that primarily impacts antibody responses, one approach to enhance vaccine efficacy may be to target T cell responses, rather than antibody responses. This would have the additional benefit of targeting responses that are likely to be more important in human infection. For example, candidate antigens that induce protective Th17 immunity may both enhance efficacy and overcome patterned antibody responses (107). In this context, toxins would be attractive candidate antigens, because they interfere with nearly every step of the host adaptive immune responses. However, the high “natural” levels of toxin-specific antibodies, many of which are cross-reactive, suggests that it will be necessary to identify protective SbD epitopes that can be used to elicit protective responses. An approach to overcome epitope masking by naturally elicited antibodies would be the design of epitope-focused vaccines that target protective but SbD epitopes (108). In order to increase the likelihood of the success of these approaches, pre-clinical studies should focus on attempting to vaccinate animals that have already been exposed to *S. aureus*, rather than reliance on naïve animals. The genetic background of experimental animals should also be considered here, since different mouse strains respond differently to *S. aureus* infection. Finally, because of the documented differences between *S. aureus* infection in mice and humans (109) and the wide-range of virulence factors that drive different infectious syndromes (110), candidate vaccines should be tested against multiple types of *S. aureus* infection, and alternative models such as rabbits and non-human primates should be considered to complement mouse studies.

### Adjuvants

Novel adjuvants that stimulate certain T cell responses may also help to overwrite patterned immunity. For example, Bagnoli et al. used a novel TLR7-dependent adjuvant to induce strong and broad protection against *S. aureus* with a multivalent vaccine including Hla, EsxA, EsxB, and the surface proteins ferric hydroxamate uptake D2 (FhuD2) and conserved staphylococcal antigen 1A (Csa1A). Importantly, they demonstrated superior protection with the TLR7 adjuvant, compared with alum (111). Monaci et al. also used MF59, an oil-in-water emulsion licensed in human vaccines, with 4C-Staph (FhuD2, Csa1A, α-Hemolysin, EsxA, and EsxB) induced stronger antigen-specific IgG titers and CD4^+^ T-cell responses compairing with alum (112). The use of novel adjuvants, perhaps in combination with epitope-focused approaches, may also improve our ability to generate antibody and T cell responses against SbD protective antigens. Given the emergence of novel adjuvants and vaccine formulations, more work is needed in this area.

### Correlates of Protection

As mentioned above, a major challenge in the development of staphylococcal vaccines is that there is a dearth of identified correlates of protection. One such example is antibody levels against Hla, which correlate with protection against recurrent infection in children (4). However, despite high anti-Hla antibody levels in children, there is some evidence that children have lower levels of neutralizing antibody. Future work should focus on identifying both serologic and cellular correlates of protection. This would enable secondary targets of vaccine efficacy, which might be particularly important in vaccinating against a relatively rare infection. Perhaps more importantly, identification of correlates of immunity will provide important mechanistic insight that can provide the foundation for future vaccine efforts. One possibility would be to determine whether *S. aureus*-specific Th17 or memory T cells can be a suitable biomarker to predict human protection against infection (113).

### Target Population

Finally, there has been much debate about the ideal target population (and infectious syndrome) for a *S. aureus* vaccine. As discussed, previous approaches have focused on populations with a high incidence of infection. However, given the high burden of *S. aureus* infection in children (114), we believe that a successful vaccine should be implemented on the population level and administered during childhood. This will have several benefits. First, this approach would leverage the childhood vaccine infrastructure and prevent infections in vulnerable populations that would not otherwise be protected. Second, if patterned immune responses prevent vaccine efficacy, vaccination prior to the onset of these responses would be anticipated to be more effective. However, this approach would require very large studies of vaccine efficacy. A corollary to this approach would be maternal vaccination. The exciting findings that vaccination of pregnant mice resulted in the protection of offspring provide pre-clinical rationale for this approach (35). However, it is appreciated that *S. aureus* is a commensal, and therefore bacterial eradication may not be possible. Future work should focus on how vaccines may prevent common infectious syndromes (e.g., skin infections) and their impact on asymptomatic colonization. It is anticipated that pragmatic application of detailed mechanistic insight will be necessary to drive the field forward.

## Conclusion

Understanding the mechanisms by which *S. aureus* evades immunological memory is critical to design a protective vaccine. As reviewed here, we believe that future studies should focus on developing strategies to circumvent the multiple mechanisms used by *S. aureus* to prevent protective T cell responses. It is anticipated that a better understanding of these host-pathogen interactions can be leveraged toward future vaccine efforts.

## Author Contributions

OT performed the literature search and drafted the manuscript. CM revised the manuscript. All authors contributed to the article and approved the submitted version.

## Conflict of Interest

The authors declare that the research was conducted in the absence of any commercial or financial relationships that could be construed as a potential conflict of interest.
